# Reproducibility and Sources of Variation of Urinary Biomarkers of Food Intake of Fruits, Vegetables, and Chocolate in European Children and Adolescents

**DOI:** 10.1016/j.tjnut.2025.10.039

**Published:** 2025-10-29

**Authors:** Jantje Goerdten, Jodi Rattner, Mira Merdas, David Achaintre, Li Yuan, Paola Russo, Toomas Veidebaum, Dénes Molnár, Lauren Lissner, Stefaan De Henauw, Luis A Moreno, Krasimira Aleksandrova, Ronja Foraita, Ute Nöthlings, Pekka Keski-Rahkonen, Anna Floegel, IDEFICS/I.Family Consortia, Jantje Goerdten, Li Yuan, Paola Russo, Toomas Veidebaum, Dénes Molnár, Lauren Lissner, Stefaan De Henauw, Luis A Moreno, Krasimira Aleksandrova, Ronja Foraita, Anna Floegel

**Affiliations:** 1 Leibniz Institute for Prevention Research and Epidemiology – BIPS, Bremen, Germany; 2 Nutrition and Metabolism Branch, International Agency for Research on Cancer (IARC/WHO), Lyon, France; 3 Department of Environmental Medicine, Icahn School of Medicine at Mount Sinai, New York, NY, United States; 4 The Institute for Exposomics Research, Icahn School of Medicine at Mount Sinai, New York, NY, United States; 5 Institute of Food Sciences, National Research Council, Avellino, Italy; 6 National Institute for Health Development, Estonian Centre of Behavioral and Health Sciences, Tallinn, Estonia; 7 Department of Pediatrics, Medical School, University of Pécs, Pécs, Hungary; 8 School of Public Health and Community Medicine, Institute of Medicine, Sahlgrenska Academy, University of Gothenburg, Gothenburg, Sweden; 9 Department of Public Health and Primary Care, Ghent University, Ghent, Belgium; 10 GENUD (Growth, Exercise, Nutrition and Development) Research Group, Faculty of Health Sciences, University of Zaragoza, Instituto Agroalimentario de Aragón (IA2) and Instituto de Investigación Sanitaria Aragón (IIS Aragón), Zaragoza, Spain; 11 Consorcio CIBER, M.P. Fisiopatología de la Obesidad y Nutrición (CIBERObn), Instituto de Salud Carlos III (ISCIII), Madrid, Spain; 12 Faculty of Human and Health Sciences, University of Bremen, Bremen, Germany; 13 Unit of Nutritional Epidemiology, Department of Nutrition and Food Sciences, Rheinische Friedrich-Wilhelms-University Bonn, Bonn, Germany; 14 Section of Nutrition and Dietetics, Faculty of Agriculture and Food Sciences, Hochschule Neubrandenburg – University of Applied Sciences, Neubrandenburg, Germany

**Keywords:** reproducibility, variability, metabolites, biomarkers of food intake, metabolomics, urine

## Abstract

**Background:**

Biomarkers of food intake may improve dietary assessment. Thereby, a key concern is their reproducibility over time. In epidemiological studies, it is important to accurately estimate habitual food intake and consequent disease risk associations.

**Objectives:**

We aimed to assess the reproducibility of 12 urinary metabolites linked to food intake and to investigate potential sources of their variation.

**Methods:**

The analyses are based on previously identified urinary metabolites associated with dietary intake of fruits, vegetables, and chocolate in the large-scale European Identification and Prevention of Dietary- and Lifestyle-Induced Health Effects in Children and Infants/I.Family study. Metabolites were measured in 1788 urine samples from 599 children at study baseline (2007/2008, *n* = 597), at the first follow-up (2009/2010, *n* = 596), and at the third follow-up (2013/2014, *n* = 595) using high-resolution liquid chromatography–MS. Unadjusted and adjusted intraclass correlation coefficients (ICCs) were calculated for 2- and 4-y intervals. To identify sources of biomarker variability, various factors, including dietary intake, were analyzed. The amount of variance explained by each factor was quantified using the partial coefficient of determination (*R*^2^).

**Results:**

The median ICCs were 0.27 (range: 0.11–0.54) and 0.28 (range: 0.15–0.51) over 2- and 4-y intervals, respectively. Individual factors explained a median of 17% (range: 9.8–42.4) of the variance for the 2-y interval and 14.6% (range: 8.3–43.8) for the 4-y interval. Country of residence explained the largest proportion of variance (median: 5% for the 2-y interval, 4.5% for the 4-y interval). Dietary intake explained only a variation of 0.7% (0.0–1.5) and 0.6% (0.0–1.1) for the 2- and 4-y interval, respectively.

**Conclusions:**

The reproducibility of urinary metabolites was poor to moderate over the 2- to 4-y periods, and only part of the variability could be explained by the studied factors. Future studies should explore shorter time intervals and other sources of variation, e.g., the influence of the gut microbiome and genetic factors.

## Introduction

Biomarkers of food intake (BFIs) can complement or enhance dietary exposure assessment [1]. BFIs are objective measures of dietary intake; however, before the biomarkers can be used in research, they must be carefully validated. One aspect that needs to be evaluated is the variability of metabolite concentrations [2].

In the present study, reproducibility denotes the variability found between 2 or more measurements made in the same subject over time, and it is quantified by the intraclass correlation coefficient (ICC). The ICC is calculated by dividing the between-person variability by the total variability (between-person and within-person variability) and can take any value between 0 and 1. An ICC value closer to 0 indicates low reproducibility, whereas an ICC value closer to 1 indicates high reproducibility [3]. Biomarkers with low ICCs introduce bias toward the null and require larger sample sizes and more repeated measurements per study participant to accurately reflect usual exposure levels [4,5]. There are several studies examining the reproducibility of blood metabolites, which show moderate-to-excellent reproducibility for short- and long-term exposure [[6], [7], [8], [9]]. Although urine is the preferred sample type for BFIs, as it is noninvasive, relatively easy to collect, and shows a good coverage of diet-related metabolites [10], there are not many studies assessing the reproducibility of urine metabolites associated with dietary intake. Wang et al. [11] investigated the reproducibility of metabolite concentrations associated with habitual dietary intake measured 6 mo apart. The overall reproducibility was moderate, with a median ICC of 0.53. Landberg et al. [12,13] calculated the ICCs for BFIs of whole grain, cereal fiber, and rye intake measured in spot urine samples. The reproducibility varied from poor to good, as ICC values ranged from 0.17 to 0.67 in the 2 studies.

The human metabolome variability is influenced by not only individual characteristics, such as age and sex, but also the individual lifestyles [14]. The proportion of variance explained by these individual characteristics can be decomposed and explored [9,15]. Investigating the magnitude of influence of different factors on the variance of metabolites can provide important insights into their future use as biomarkers. Two recent studies have investigated sources of variation in adult populations [9,14]. Li-Gao et al. [9] calculated the proportion of variance explained by age, sex, dietary state (fasting or postprandial), time interval (short or long term), and between- and within-individual variability. Only a small proportion of variation could be explained by sex and age; however, ∼66% was due to between-subject variability. In the study by Rafiq et al. [14], the influence of dietary factors was assessed. They found that only a small amount of the variance (<12%) of metabolite concentrations associated with dietary intakes is explained by dietary factors. Lau et al. [16] investigated the explained variance of urinary metabolic profiles in children. Although dietary factors accounted for the largest proportion of explained variance, the median was 1.6%.

So far, only 1 study has investigated the sources of variation of BFIs in European children but not its reproducibility [16]. The reproducibility and the sources of variation of 12 previously identified urinary metabolites associated with dietary intake of fruits, vegetables, and chocolate in European children and adolescents are unknown [17]. Hence, the aim of this work was 2-fold: *1*) to assess the reproducibility of previously identified urine metabolites as BFIs of chocolate, fruits, and vegetables in European children over 2- and 4-y intervals and *2*) to investigate potential sources of variability.

## Methods

### Study population

The analyses were based on data from the “Identification and Prevention of Dietary- and Lifestyle-Induced Health Effects in Children and Infants” (IDEFICS) and I.Family cohort. The study aimed to assess risks of overweight and obesity in European children and associated long-term effects. The cohort comprises children and adolescents from 8 European countries: Belgium, Cyprus, Estonia, Germany, Hungary, Italy, Spain, and Sweden. They were assessed repeatedly at baseline and 5 follow-up time points. Recruitment was carried out in kindergarten and school settings. Baseline examinations were rolled out between 2007 and 2008. A total of 16,228 children aged between 2 and 9 y were examined at baseline. The cohort was described in detail elsewhere [[18], [19], [20], [21]]. The study protocol was approved by the appropriate ethics committees from each of the 8 study centers. The parents gave written informed consent.

**FIGURE 1:**
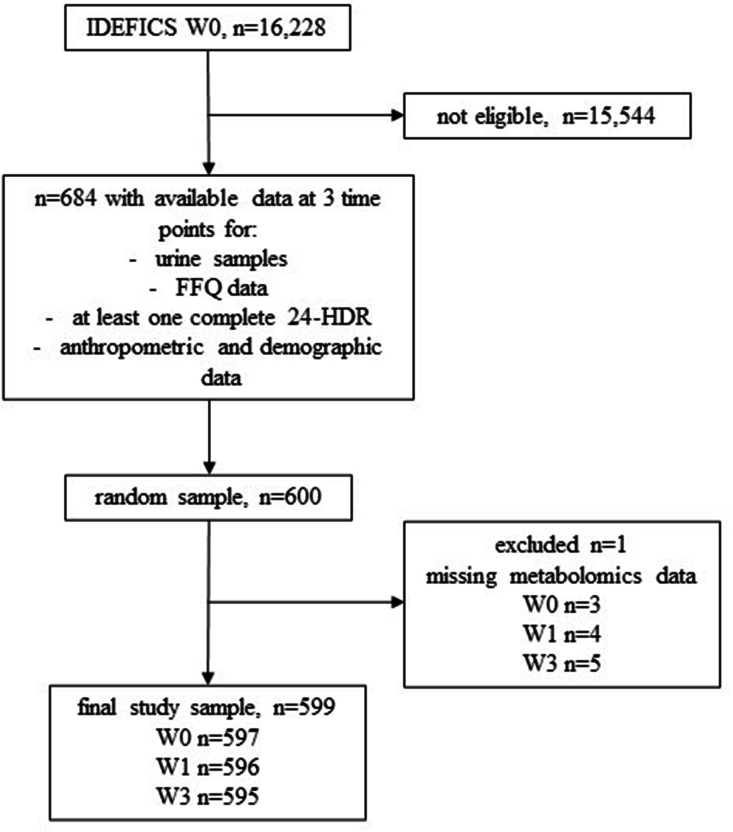
Flow diagram of the sample selection. FFQ, Food Frequency Questionnaire; IDEFICS, Identification and Prevention of Dietary- and Lifestyle-Induced Health Effects in Children and Infants; 24-HDR, 24-h dietary recall.

From the initial cohort, 600 children and adolescents were randomly selected who had urine samples and dietary information available at 3 time points—in the periods from 2007 to 2008 (W0), 2009 to 2010 (W1), and 2013 to 2014 (W3), respectively. Excluding missing laboratory data, the sample size of the analytical data set was reduced to *n* = 599 children with *n* = 1788 urine samples available (W0: *n* = 597, W1: *n* = 596, and W3: *n* = 595). For a flow diagram of the study sample selection, see Figure 1. The sample was divided into 2-y (W0 and W1) and 4-y (W1 and W3) time intervals between metabolite measurements. This approach allowed us to reduce the total time span between measurements (compared with using W0 and W3, which would result in a 6-y time interval), while leveraging the full longitudinal data set.

### Assessment of covariates

Individual characteristics, such as biological sex, age, and country of residence, were recorded at each time point. Questionnaires were administered at each time point, in which the highest educational level of both parents according to the International Standard Classification of Education (ISCED) was documented. Anthropometric data were routinely collected over each follow-up time point. Well-trained study nurses measured the height, weight, and waist circumference of the participants [22]. The BMI was calculated by dividing the weight by the squared height (kg/m^2^), and the waist-to-height ratio (WHR) was computed by dividing the waist measurement by the height measurement. Physical activity was measured by Actigraph accelerometers (Actigraph, LLC) worn on consecutive days by the study participants [23]. Clinical biomarkers such as glucose, C-reactive protein (CRP), HDL-cholesterol, and triglycerides (TRGs), were measured in fasting venous blood collected from the children. For a more in-depth description of biological sample collection, storage, and laboratory analyses, see the study by Peplies et al. [24].

### Dietary intake assessment

The dietary intake of children and adolescents was recorded by computer-based 24-h dietary recalls (24-HDRs) with the “Self-Administered Children and Infant Nutrition Assessment” program at W0 and W1 and the Web-based 24-HDR with the “Self-Administered Children, Adolescents, and Adult Nutrition Assessment” program at W3 [25]. Additionally, the Children’s Eating Habits Questionnaire—Food Frequency Questionnaire (FFQ) was administered at each follow-up time point [26]. For more information about the 24-HDR and FFQ, see Supplemental Material.

The short-term dietary intake was computed by summing the intakes (in grams) measured from the 24-HDR. As the urine collection and short-term dietary assessment did not match for all study participants, the sample size for the short-term intake variables was *n* = 444, W0: *n* = 116, W1: *n* = 105, and W3: *n* = 223. For more information, see the study by Goerdten et al. [17].

To estimate the habitual dietary intake (grams/day), the method developed by the National Cancer Institute (NCI) was applied [27]. Data from the 24-HDR and FFQ from each examination were used in the estimation. The analysis was adjusted for age and BMI *z*-score and stratified by sex.

Ultimately, short-term chocolate, short-term apple, short-term orange, and short-term potato intake, and habitual vegetable, habitual fruit, and habitual chocolate intake were derived.

### Biomarkers of food intake

Twelve BFIs were previously identified to be (putative) markers of fruit, vegetable, and chocolate intake in children and adolescents and were externally replicated [17]. The following metabolites were selected: theobromine, xanthosine, cyclo(L-prolyl-L-valyl), octenoylcarnitine, 5-hydroxyindoleacetic acid (5-HIAA), D-pantothenic acid, hippuric acid, ferulic acid 4-O-glucuronide, ferulic acid 4-O-sulfate, and gentisic acid. These BFIs were measured in urine samples routinely collected during the IDEFICS/I.Family study [24]. Urine samples were stored at the study centers and shipped to the central biorepository at regular intervals. From the main biorepository, the 1800 urine samples were shipped to the laboratory at the International Agency for Research on Cancer (IARC, Lyon, France), where the urine samples were analyzed by liquid chromatography–MS–based untargeted metabolomics as previously described [17]. The MS analysis was analyzed in polarity-switching electrospray ionization mode. The samples were analyzed in 10 independent batches, with sample pairs randomly assigned across the batch and repeated samples next to each other. The mean storage time between collection and metabolomic analysis was ∼14 y (W0), 12 y (W1), and 8 y (W3). The metabolite annotation was also carried out at the IARC. For a more detailed description of the untargeted metabolomics analysis, see Supplemental Material, and for an overview of the response variability of the quality controls, see Supplemental Figures 1 and 2 in the Supplemental Material. A brief description of the metabolites and their identification are also provided in the Supplemental Material and Supplemental Table 1.

### Statistical analyses

This study is a secondary data analysis based on information collected in the IDEFICS/I.Family cohort. All analyses were carried out in R (version 4.2.2) [28] and RStudio (version 2022.07.2+576) using the following central packages: mice (version 3.16.0) [29], lme4 (version 1.1.30) [30], performance (version 0.10.4) [31], and partR2 (version 0.9.1) [32].

The values of BMI, WHR, glucose, CRP, HDL, and TRG were *z-*standardized according to age and sex. The BFIs were log transformed and *z*-standardized before analysis. Multiple imputations (*R* = 20) were performed for WHR *z-*score, glucose *z*-score, CRP *z*-score, HDL *z*-score, TRG *z*-score, and physical activity. A complete-case sensitivity analysis was performed (data not shown). For an overview of the missing data structure (Supplemental Table 2) and details about the imputation procedure, see Supplemental Material and Supplemental Table 3. The covariates were selected to capture individual characteristics that might influence the metabolome and therefore the variability of the measured BFIs [33,34].

For the reproducibility analysis, linear mixed models with the BFIs as the outcome variable, the subject ID number as a random effect, and (A) no other covariate and (B) age, sex, and country were computed. The ICC was calculated for each model by dividing the random effect variance by the total variance (the sum of the random effect variance and the residual variance). The ICC was interpreted as <0.4 poor, ≥0.4 moderate, ≥0.7 good, and ≥0.85 excellent reproducibility [3].

For the analyses of sources of variation, a linear mixed model was fitted with the BFIs as the outcome variable; subject ID number as the random effect; and dietary intake, sex, age, country, time point, BMI *z*-score, energy intake, ISCED, batch, season of urine collection, physical activity, WHR *z*-score, CRP *z*-score, glucose *z*-score, HDL *z*-score, and TRG *z*-score as the covariates. The explained variance, i.e., partial coefficient of determination (*R*^2^) for each covariate was extracted from the model using the function “partR2.” The partial *R*^2^ is computed by assessing the reduction in the fixed-effect variance that occurs when a covariate or a group of covariates is excluded from the model, compared with the total estimated variance [32]. Bootstrap samples (*R* = 500) were used to determine 95% confidence intervals for the *R*^2^ values. A sensitivity analysis was conducted to evaluate whether the imputation had any impact on the results. In the sensitivity analysis, only participants without any missing values for WHR *z*-score, glucose *z*-score, CRP *z*-score, HDL *z*-score, TRG *z*-score, and physical activity were included (*n* = 514, W0: *n* = 24, W1: *n* = 454, and W3: *n* = 272; results not shown).

## Results

Table 1 shows the baseline and follow-up characteristics of the study sample. The study sample consisted of 47% girls. The median age of the children was 6.4 y at baseline and 12.3 y at the second follow-up. Most of the children came from Italy, Estonia, or Germany. Furthermore, most of the children had parents with middle or high educational backgrounds. Usual chocolate intake increased from baseline to the follow-up time points, whereas total fruit intake and vegetable intake decreased. Median levels of the urinary metabolites associated with chocolate, fruit, and vegetable intake are reported in Supplemental Table 4.

**TABLE 1:** Characteristics of the main study sample at each examination wave

	W0 *N* = 597	W1 *N* = 596	W3 *N* = 595
	Median (range) or *n* (%)		
Age (y)	6.4 (2.1–9.3)	8.4 (4.0–11.1)	12.3 (8.0–15.2)
Female	281 (47%)	282 (47%)	280 (47%)
Country			
Italy	288 (48%)	287 (48%)	288 (48%)
Estonia	140 (23%)	140 (23%)	139 (23%)
Belgium	12 (2%)	12 (2%)	12 (2%)
Sweden	51 (9%)	50 (8%)	49 (8%)
Germany	62 (10%)	63 (11%)	63 (11%)
Hungary	30 (5%)	30 (5%)	30 (5%)
Spain	14 (2%)	14 (2%)	14 (2%)
ISCED class			
Low	48 (8%)	46 (8%)	48 (8%)
Middle	295 (49%)	279 (47%)	278 (47%)
High	254 (43%)	271 (45%)	269 (45%)
PA (h/wk)	14.8 (0–56)	16 (0.5–70)	15.5 (0–62)
BMI *z*-score	0.42 (−2.79–5.07)	0.48 (−3.3, 4.18)	0.66 (−2.13, 3.64)
Season of urine collection			
October–December	179 (30%)	225 (38%)	181 (30%)
January–March	266 (45%)	245 (41%)	211 (36%)
April–June	147 (25%)	124 (21%)	183 (31%)
July–September	5 (1%)	2 (0.3)	20 (3%)
WHR *z*-score	0.21 (−3.2, 5)	0.42 (−3.43, 5)	0.37 (−2.93, 3.94)
CRP *z*-score	0.22 (−3.11, 3.23)	0.22 (−1.11, 3.41)	0.02 (−3.53, 2.64)
Glucose *z*-score	0.29 (−2.82, 3.599)	0.03 (−2.93, 3.94)	0.08 (−3.78, 4.27)
HDL *z*-score	−0.03 (−3.07, 2.78)	−0.2 (−3.44, 2.92)	−0.07 (−2.4, 2.82)
Triglycerides *z*-score	−0.58 (−2.77, 2.86)	0.26 (−1.21, 3.18)	−0.01 (−5.0, 2.86)
Energy intake	1642 (1136–2409)	1613 (900–2802)	1643 (779–2656)
habitual chocolate intake (g/d)	7.8 (1.2–55.6)	6.8 (2.1–52.2)	11.4 (2.7–71.2)
habitual fruit intake (g/d)	165.0 (6.0–484.9)	185.0 (7.2–448.4)	145.0 (42.0–506.8)
habitual vegetable intake (g/d)	109.5 (36.3–318.9)	121.2 (4.9–407.3)	104.4 (37.0–356.4)
Chocolate intake (g/d)	0 (0–200)	0 (0–150)	0 (0–634)
Orange intake (g/d)	0 (0–800)	0 (0–1100)	0 (0–850)
Apple intake (g/d)	0 (0–1075)	0 (0–600)	0 (0–11,177)
Potato intake (g/d)	0 (0–380)	0 (0–500)	0 (0–725)

Abbreviations: ISCED, International Standard Classification of Education; PA, physical activity; WHR, waist-to-height ratio; CRP, c-reactive protein.

### Reproducibility

The 2-y and 4-y ICCs for 12 urinary metabolites associated with fruit, vegetable, or chocolate intake in our sample are presented in Table 2. The 2-y unadjusted ICC ranged from 0.11 to 0.54, and the 4-y unadjusted ICC ranged from 0.15 to 0.51. Octenoylcarnitine, which was positively associated with vegetable intake, had the highest ICCs with 0.54 and 0.51 for the 2-y and 4-y intervals, respectively. Moderate reproducibility over 2 y was also found for D-pantothenic acid, which was positively linked to total fruit intake, and hippuric acid, which was positively associated with apple intake as well as total fruit and vegetable intake. The other 9 metabolites that were linked to the intake of chocolate, fruits, or vegetables showed poor reproducibility with ICCs below 0.4. Adjusting for age and sex resulted in slight changes (increase and decrease) for the 2-y ICCs and an overall slight increase in the ICC values for the 4-y interval.

**TABLE 2:** Intraclass correlation coefficients computed from linear mixed models for the dietary biomarkers with 2- and 4-y measurement intervals

Metabolite	Food intake^1^	2-y		4-y	
		ICC	ICC adjusted^2^	ICC	ICC adjusted^2^
Theobromine	habitual chocolate	0.34	0.32	0.30	0.29
Xanthosine^3^	short-term chocolate	0.26	0.25	0.29	0.30
Xanthosine^4^	habitual chocolate	0.23	0.23	0.26	0.29
Cyclo(L-prolyl-L-valyl)	habitual chocolate	0.26	0.26	0.26	0.27
Octenoylcarnitine	habitual vegetables	0.54	0.55	0.51	0.53
5-HIAA	habitual fruits	0.11	0.14	NA^5^	0.09
D-Pantothenic acid	habitual fruits	0.40	0.40	0.33	0.34
Hippuric acid^3^	habitual vegetables	0.37	0.37	0.31	0.31
Hippuric acid^4^	short-term apple	0.40	0.40	0.28	0.28
Ferulic acid 4-O-glucuronide	short-term orange	0.28	0.27	0.24	0.24
Ferulic acid 4-O-sulfate	short-term orange	0.22	0.21	0.25	0.25
Gentisic acid	short-term potato	0.11	0.11	0.15	0.17

1 Association with food intake; short-term: dietary intake 1 or 2 d before urine collection and *n* = 444; habitual: dietary intake calculated with the United States: National Cancer Institute (NCI) method and *n* = 599.

2 Adjusted for age and sex.

3 Measured in positive ionization mode.

4 Measured in negative ionization mode.

5 Linear mixed model is singular, could not compute.

### Sources of variation

**FIGURE 2:**
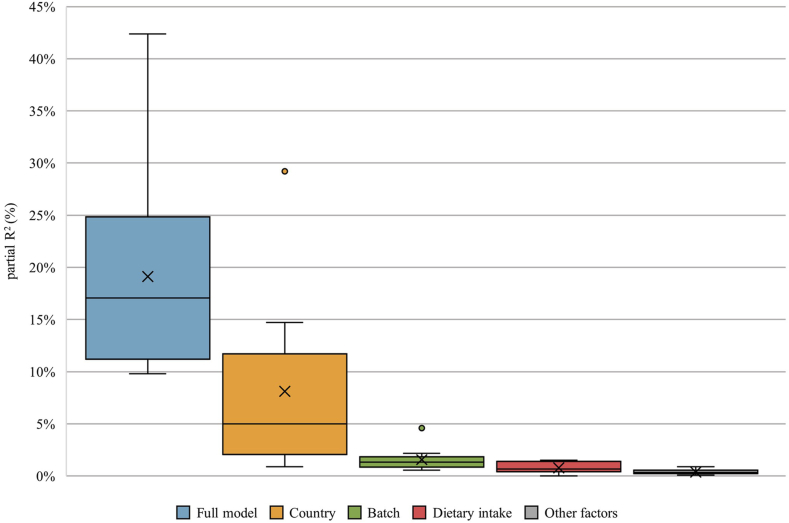
Boxplots showing the partial *R*^2^ values for all 12 metabolites of the main factors measured 2 y apart. Full model: variance explained by all factors combined (dietary intake, sex, age, country, time point, BMI *z*-score, energy intake, International Standard Classification of Education (ISCED), batch, season of urine collection, physical activity, waist-to-height ratio (WHR) *z*-score, C-reactive protein (CRP) *z*-score, glucose *z*-score, HDL *z*-score, and triglyceride [TRG] *z*-score). Country: decomposed variance explained by country. Batch: decomposed variance explained by batch. Dietary intake: decomposed variance explained by dietary intake (dependent on each metabolite, see Table 2). Other factors: mean decomposed variance explained by sex, age, time point, BMI *z*-score, energy intake, ISCED, season of urine collection, physical activity, WHR *z*-score, CRP *z*-score, glucose *z*-score, HDL *z*-score, and TRG *z*-score.

**FIGURE 3:**
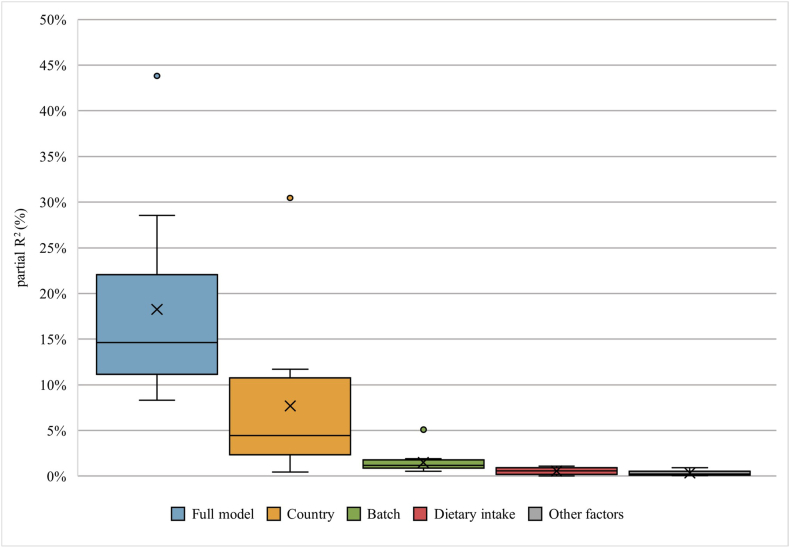
Boxplots showing the partial R^2^ values for all 12 metabolites of the main factors measured 4 years apart. Full model: variance explained by all factors combined (dietary intake, sex, age, country, time point, BMI *z*-score, energy intake, International Standard Classification of Education (ISCED), batch, season of urine collection, physical activity, waist-to-height ratio (WHR) *z*-score, C-reactive protein (CRP) *z*-score, glucose *z*-score, HDL *z*-score, and TRG z-score). Country: decomposed variance explained by country. Batch: decomposed variance explained by batch. Dietary intake: decomposed variance explained by dietary intake (dependent on each metabolite, see Table 2). Other factors: mean decomposed variance explained by sex, age, time point, BMI *z*-score, energy intake, ISCED, season of urine collection, physical activity, WHR *z*-score, CRP *z*-score, glucose *z*-score, HDL *z*-score, and TRG *z*-score.

The partial *R*^2^ values are presented in FIGURE 2, FIGURE 3 for the 2-y and 4-y interval, respectively. The median 2-y partial *R*^2^ for the whole set of individual factors was 17% (range: 9.8–42.4), and the median 4-y partial *R*^2^ for the whole set of individual factors was 14.6% (range: 8.3–43.8). Country explained most of the variance for the 2-y (median: 5%; range: 0.9–29.2) and 4-y interval (median: 4.5%; range: 0.5–30.5) in the models. Dietary factors only explained a very small proportion of the variance in metabolites measured 2 (median: 0.7%; range: 0.0–1.5) and 4 y (median: 0.6%; range: 0.0–1.1) apart. Most of the variance could be explained for the metabolite octenoylcarnitine, with >40% for both time intervals. In contrast, the least variance was explained for the metabolites gentisic acid and ferulic acid 4-O-sulfate, with <10% for the 2-y and 4-y intervals. Octenoylcarnitine also had the highest ICC values and gentisic acid had the lowest for the 2-y interval. Interestingly, for gentisic acid, which was linked to short-term potato intake, the dietary intake contributed the most to the explained variance, with 1.1% and 1.5% for the 2-y and 4-y intervals, respectively. The *R*^2^ values for each individual factor for the 12 metabolites measured 2 and 4 y apart are presented in Supplemental Tables 5–28. The complete-case sensitivity analysis showed no detectable effect on the results (data not shown).

## Discussion

In the present study, the reproducibility and the sources of variation of 12 newly identified BFIs in children were assessed. The reproducibility of octenoylcarnitine was moderate, and for all other BFIs, the reproducibility was poor, over this period of several years. The dietary intake only explained a small amount of the variation between metabolite measurements. The results add to the validation of these potential candidate biomarkers and further to the understanding of the variations seen in urinary metabolomic measurements.

To the best of our knowledge, there are no other studies investigating the reproducibility of the metabolites studied here. However, 3 studies investigated the reproducibility of other urinary BFIs. The findings ranged from ICCs of 0.17 to 0.67 [[11], [12], [13]]. Differences can be explained by the varying intervals between sample collections in individual studies. In the study by Landberg et al. [13], the authors analyzed 4 urine samples collected over a period of 2 wk. Overall, the reproducibility ranged from moderate to excellent (ICCs: 0.35–0.67). However, in another study, Landberg et al. [12] analyzed 2 urine samples collected 1 and 3 y apart, where the reproducibility was overall poor (ICCs: 0.17 and 0.31). In a study by Wang et al. [11], which was based on urine sample collections 6 mo apart, the overall reproducibility was moderate (median ICC: 0.53 and interquartile range: 0.42–0.62). Our study is in line with previous studies considering the longer time interval of ≤4 y. However, the long-term reproducibility of the dietary biomarkers can be classified as mostly poor. The findings of the current study indicate that, to accurately reflect exposure, multiple measurements per subject are needed when habitual intake is studied. The lower the ICC value, the higher the number of measurements needed. Perrier et al. [4] estimated that 6–35 samples would be required per subject if the ICC was 0.6 or 0.2, respectively. They also found significant bias introduced by biomarkers with a low ICC value. An ICC value of 0.11, as found for 5-HIAA or gentisic acid, would attenuate a true relative risk of 1.8 to an observed relative risk of 1.07 [35]. However, as stated by Cuparencu et al. [36], a low ICC value for a BFI does not necessarily indicate that it cannot be used in future research, but it does suggest limitations in its use. Some metabolites may not be suitable as BFIs in epidemiological studies unless further research can demonstrate their robustness. There are 3 possible explanations for a low ICC value: a short time window of sampling, i.e., the BFI has a short half-life of elimination, the corresponding food is consumed infrequently, and/or the response of the individuals varies considerably over time. The ICC value is affected by the half-life of elimination, exposure frequency, and other biological factors, such as absorption and metabolism [3,36]. The variability of BFI measurements is the highest when the half-life is short and the dietary intake is infrequent [37]. If a biomarker has a short half-life, the window for sampling is small, and if the food is consumed infrequently, the likelihood of measuring the BFI is low [36,37]. In our previous study [17], we included only foods that were frequently consumed by children. Unfortunately, the half-life of elimination is unknown for most of the BFIs investigated in the present study. Interestingly, Aylward et al. [37] reported that the half-life of a biomarker also depends on the individual; in 1 person, the biomarker may have a shorter or longer half-life. Additional studies with repeated short-term measurements may be required to validate the use of these metabolites as reliable BFIs.

In the present study, we investigated possible sources of variation for the 12 urinary metabolites. The results provide insight into the factors contributing to both within- and between-individual variability in metabolite measurements. Ideally, we would expect the variation in a dietary metabolite to be primarily driven by dietary intake, i.e., the exposure the metabolite is intended to reflect. However, our findings indicate that dietary intake accounts for only a small fraction of the explained variance. Most of the variance is explained by country of residence. Fages et al. [15] applied a similar analysis to data from the European Prospective Investigation into Cancer and Nutrition study. The results show that country explains most of the variance found in serum metabolites. In a study of European children, dietary information contributed the largest proportion of explained variance (median: 1.6%), closely followed by cohort origin, ethnicity, and batch (median: 1.5%) [16]. Indeed, batch is also found in our study as an important source of variation (median: 1.3% and 1.2%), although in a much smaller proportion than country. In the study by Lau et al. [16], individual factors explain more of the variance found in serum metabolites than in urine metabolites. Similarly, the individual factors included in the present study, including batch, only explain ∼19% of the variance on mean. This raises the question of what else could be driving the variance of these urinary metabolites. Technical factors such as sample collection, storage, preparation, and analysis have been shown to contribute to the variability seen in metabolite measurements [38]. Indeed, the storage time between collection and laboratory analysis was several years, which potentially resulted in chemical alterations [39]. In addition to technical factors related to sample handling or storage, the intrinsic physicochemical properties of metabolites, such as susceptibility to oxidation, hydrolysis, or degradation, can also affect their stability during laboratory analyses. In the present study, we accounted for batch and season of urine collection as reflections of technical factors. Technical variability, especially batch effects, is more pronounced in untargeted metabolomics analyses. The use of targeted approaches could potentially reduce this type of variability. Considering technical factors may help explain a larger part of the observed variability. Moreover, the biological variability of metabolite levels in urine is affected by the gut microbiota and the genetics, which should be considered in future variability research [36].

The limitations of the present study are the following: This research relies on secondary data from the IDEFICS/I.Family cohort, which was not specifically designed to address the objectives of this study. The storage time of the urine samples at the central biorepository was long and varied between study waves, which might have influenced the observed variability. Moreover, there were missing values for most of the individual factors included in the sources of variation analysis. The potential influence of imputed values could be in both directions, i.e., toward and away from the null. However, the complete-case analysis showed no detectable effect on the results. The present study had several strengths. We used a large cohort of European children and adolescents. However, almost half of the children were from 1 country, which was due to the biosample availability. The biosamples were measured at 3 time points, which enabled us to investigate 2 different time intervals, i.e., 2-y and 4-y intervals. The application of the NCI method enabled us to combine the data from the FFQ and 24-HDR, which allowed for a precise calculation of the habitual intake [40]. The 12 metabolites were measured in urine, a noninvasive biological matrix particularly suitable for children and adolescents, which can reflect both short-term and habitual dietary intake [36].

In the present study, we assessed the reproducibility and sources of variation for 12 urinary metabolites, a crucial step toward evaluating their potential application as biomarkers in future epidemiological studies. However, further investigation is needed to evaluate their reproducibility over shorter periods, such as days, weeks, or months. Furthermore, future studies should consider shorter storage times of the biospecimen. In addition, because only a small fraction of the biological variability could be explained, more research into potential influencing factors is warranted. In particular, gut microbiome composition and genetic variation are promising areas to explore in future analyses. Moreover, future studies should consider a broader range of metabolites and alternative biological media, such as plasma, to determine whether these may offer improved long-term stability compared with urine. This could help identify more robust biomarkers for use in nutritional epidemiology and related fields.

## Author contributions

The authors’ responsibilities were as follows—AF, UN, PK-R, JR: designed research; PR, TV, DM, LL, SDH, LAM: authors conducted research; JR, MM, DA, PK-R: performed and oversaw laboratory analyses, raw data preprocessing and metabolite annotation; LY: provided essential materials; JG analyzed data and performed the statistical analysis; RF: provided guidance on the statistical analysis; JG: wrote paper; AF, KA: supervised and made significant contributions to the writing process of the paper; JG, AF: had primary responsibility for final content; and all authors read and approved the final manuscript.

## Data availability

Data described in the manuscript, code book, and analytic code will be made available upon request pending application and approval.

## Funding

This study was funded by the German Research Foundation (DFG project number 406710821) and the Agence Nationale de la Recherche (ANR project number ANR-18-CE92-0060). This work was done as part of the IDEFICS (http://www.idefics.eu) and I.Family studies (http://www.ifamilystudy.eu/). We gratefully acknowledge the financial support of the European Commission within the Sixth RTD Framework Programme Contract No. 016181 (FOOD), and the Seventh RTD Framework Programme Contract No. 266044.

## Conflict of interest

AF reports financial support by German Research Foundation. PK-R reports financial support by French National Research Agency. Where authors are identified as personnel of the International Agency for Research on Cancer/WHO, the authors alone are responsible for the views expressed in this article, and they do not necessarily represent the decisions, policy, or views of the International Agency for Research on Cancer /WHO. All other authors declare no potential conflicts of interest.
