# Supplementary material for: Reproducibility and Sources of Variation of Urinary Biomarkers of Food Intake of Fruits, Vegetables, and Chocolate in European Children and Adolescents
Source: J Nutr. 2025 Oct 29;156(1):101226. doi: 10.1016/j.tjnut.2025.10.039 (PMC12881672; doi:10.1016/j.tjnut.2025.10.039)
Supplement: Multimedia component 1 [file mmc1.docx]

**Supplementary Material**

**Reproducibility and sources of variation of urinary biomarkers of food intake of fruits, vegetables and chocolate in European children and adolescents**

Jantje Goerdten^1*^, Jodi Rattner^2^, Mira Merdas^2^, David Achaintre^2,3,4^, Li Yuan^1*^, Paola Russo^5*^, Toomas Veidebaum^6*^, Dénes Molnár^7*^, Lauren Lissner^8*^, Stefaan De Henauw^9*^, Luis A. Moreno^10,11*^, Krasimira Aleksandrova^1,12*^, Ronja Foraita^1*^, Ute Nöthlings^13^, Pekka Keski-Rahkonen^2^, Anna Floegel^1,14*^

^1^Leibniz Institute for Prevention Research and Epidemiology – BIPS, Bremen, Germany

^2^International Agency for Research on Cancer (IARC), Lyon, France

^3^Department of Environmental Medicine, Icahn School of Medicine at Mount Sinai, New York, New York, USA

^4^The Institute for Exposomics Research, Icahn School of Medicine at Mount Sinai, New York, New York, USA

^5^Institute of Food Sciences, CNR, Avellino-Italy

^6^National Institute for Health Development, Tallinn, Estonia

^7^Department of Pediatrics, Medical School, University of Pécs, Pécs, Hungary

^8^School of Public Health and Community Medicine, Institute of Medicine, Sahlgrenska Academy, University of Gothenburg, Gothenburg, Sweden

^9^Department of Public Health and Primary Care, Ghent University, Ghent, Belgium

^10^GENUD (Growth, Exercise, NUtrition and Development) Research Group, Faculty of Health Sciences, University of Zaragoza, Instituto Agroalimentario de Aragón (IA2) and Instituto de Investigación Sanitaria Aragón (IIS Aragón), Zaragoza, Spain

^11^Consorcio CIBER, M.P. Fisiopatología de la Obesidad y Nutrición (CIBERObn), Instituto de Salud Carlos III (ISCIII), Madrid, Spain

^12^Faculty of Human and Health Sciences, University of Bremen, Bremen, Germany

^13^Unit of Nutritional Epidemiology, Department of Nutrition and Food Sciences, Rheinische Friedrich-Wilhelms-University Bonn, Bonn, Germany

^14^Section of Nutrition and Dietetics, Faculty of Agriculture and Food Sciences, Hochschule Neubrandenburg - University of Applied Sciences, Neubrandenburg, Germany

* On behalf of the IDEFICS/I.Family Consortia

**24-Hour Dietary Recall (24HDR) and Food Frequency Questionnaire (FFQ)**

Study participants were asked to complete at least two weekday 24-HDRs and one on the weekend; if this was not fulfilled, participants were reminded by telephone or email [1]. The 24-HDR comprised the dietary intake (type and amount of foods and beverages) of the last 24 hours [2]. The intakes were structured into six meal occasions, namely breakfast (first intake after waking up), mid-morning snack, lunch, afternoon snack, dinner, and evening snack, while more snack occasions could be added. Quantities of foods were estimated using standardized photographs of serving sizes, standard portions, customary packaging sizes and meals in pieces or slices. For the 24-HDR based on the SACINA program, a proxy respondent, i.e., primary caretaker of the child, recalled the dietary intakes. Otherwise, the child or adolescent was asked to recall the dietary intake of the last 24 hours themselves or with the help of the primary caretaker. At each 24-HDR, a dietician or trained study nurse was present for any questions [3]. Several efforts have been made to validate the 24-HDR in the IDEFICS/I.Family cohort, for more information, see Hebestreit, Wolters [4].

The FFQ comprised 60 food items grouped into 15 food groups, specifically vegetables, fruits, breakfast cereals, milk, yoghurt, cheese, fish, meat and meat products, eggs, meat replacement products and soy products, spreadable products, cereal products and snacks, plant oil, and beverages [5]. Possible answers for the FFQ section were “never/less than once a week”, “1–3 times a week”, “4–6 times a week”, “1 time/day”, “2 times a day”, “3 times a day”, and “I have no idea”. The previous month was set as a reference for the FFQ. During W0 proxy respondents, i.e., primary caretaker of the child, answered for the child, while in W1 and W3, children and adolescents answered the FFQ section themselves. The measured food consumption of the FFQ was validated against nutrients measured in blood and urine [6].

**Sample Preparation**

Samples (n=1800) were prepared by diluting 30 µL of urine with ultra-pure water based on normalization to the lowest specific gravity in all samples (IDEFICS: 1.008). Then, 30 µL of the diluted urine samples were mixed with 270 µL of cold acetonitrile in Agilent Captiva 96 Deep Well plates (Agilent Technologies France; ref: A696001000B). The precipitate was filtered and 100 µL was transferred to Thermo Well 96 plates (Thermo Electron SAS; ref: 6820-4100). The plate was immediately sealed with a rapid EPS adhesive plate sheet (Teknolab Sorbent;ref: BC-REPS001) and analysed. Quality control (QC) samples were prepared from a sample pool that was made by mixing small aliquots of all samples and extracted along with the study samples. Blank samples were also prepared along with the urine samples in an identical manner, only leaving out the urine in the process. Each well plate included four individually prepared QCs and two blanks.

**Sample Analysis**

Samples were analysed as ten independent analytical batches consisting of two individual 96-well plates. The repeated samples were analysed next to each other in random order, and sample pairs were randomized across the batch. The analysis was performed using a UHPLC-QE-MS system that consisted of a Dionex UltiMate 3000 Binary LC system and a Q-Exactive mass spectrometer with heated electrospray ionization (HESI-II) (Thermo Scientific). Samples were kept at 5°C and 2 µL was injected. An ACQUITY UHPLC HSS T3 column (2.1 × 100mm, 1.8 μm; Waters) was used at 45 °C and the mobile phase consisted of ultrapure water and LC-MS grade methanol, both with 0.05 % (v/v) of formic acid. The gradient profile was as follows: 0–6 min: 5% to 100% methanol, 6–10.5 min: 100% methanol, 10.5–13 min: 5% methanol. The flow rate was 0.4 ml/min.

The mass spectrometer was operated in a positive/negative switching polarity using the following conditions: spray voltage 4.0 kV, sheath gas flow rate 50 (Arbitrary unit; A.u), auxillary gas flow rate 13 (A.u), sweep gas flow rate 3 (A.u), Aux gas heater temperature 425°C, capillary temperature 260°C and a S-Lens RF level 60%. For the analysis, a full MS scan mode over a mass range of 66.7 to 1000 Da, at a resolution of 35000, with an associated scan rate at 2.1 Hz was employed. AGC target 1^e6^ and a maximum injection time of 50 ms was applied. MS/MS analyses were performed on QC samples with an isolation width of 2.0 Da, in positive and negative modes at 3 normalized collision energies 30, 60 and 90. Data was acquired in centroid format.

**Data Processing**

Pre-processing was performed using Compound Discoverer 3.3 software (Thermo Fisher Scientific). A minimum peak intensity threshold and mass tolerance of 500,000 and 5 ppm, respectively, were used to find [M+H]^+^ and [M-H]^-^ ions in positive and negative mode data, respectively. Feature alignment between samples was performed with a maximum retention time window of 0.05 min and mass tolerance of 5 ppm. Features were put forward into the feature table only if they were present in at least 2% of the overall samples. Features present in every blank sample were excluded, unless 5-fold greater in average intensity in samples. The final feature table was exported as a .xlsx file, with FoodMetch ID (FM_ID) on row 1, acquisition order (Study_ID) on row 2, specific gravity measurement (SPG) in row 3, dilution factor (Dilution_Factor) in row 4, batch assignment (Batch) on row 5, and feature IDs in column 1 from row 7 onwards. Peak areas were used as a measurement of intensity.

**Quality Control**

Quality control was performed using data from the QC samples. The assessment was based on the following attributes:

- Response stability: in chronological order, area median response of features found in all QC samples
- Response variability: distribution of MS features according to their Relative Standard Deviations (RSD%) of features found in all QC samples
- Response variability: RSD% of 10 known compounds in all QC samples

**Supplementary Figure 1** A variability histogram for the features found in Positive Ionization mode of all QC samples in IDEFICS analysis (n=80).

Each batch contained two plates, and each plate contained four QC samples.

**Supplementary Figure 2** A variability histogram for the features found in Negative Ionization mode of all QC samples in IDEFICS analysis (n=80).

Each batch contained two plates, and each plate contained four QC samples.

**Biomarkers of food intake**

Theobromine, xanthosine, cyclo(L-prolyl-L-valyl), octenoylcarnitine, 5-HIAA, D-Pantothenic acid, hippuric acid, ferulic acid 4-O-glucuronide, ferulic acid 4-O-sulfate and gentisic acid were previously identified in the IDEFICS/I.Family study. The statistical analyses of the identification, as well as the food groups, for theobromine, xanthosine and cyclo(L-prolyl-L-valyl) are described in detail in Goerdten et al. 2024 [7]. In brief a two-step approach consisting of machine learning algorithms and linear mixed models were applied to identify these biomarkers. Additionally, the results were externally replicated in the Dortmund Nutritional and Anthropometric Longitudinal Designed (DONALD) cohort [8]. Octenoylcarnitine, 5-HIAA, D-Pantothenic acid, hippuric acid, ferulic acid 4-O-glucuronide, ferulic acid 4-O-sulfate and gentisic acid were similarly identified and replicated, however these results are not yet published.

**Supplementary Table 1 overview of identified metabolites and associated food intake**

| **Metabolites** | **Food group** | **Intake type**^1^ | **HMDB ID** | **Replication in DONALD** |
| --- | --- | --- | --- | --- |
| Theobromine | Chocolate candy | Short-term  Habitual | 0002825 | Yes |
| Xanthosine^2^ | Chocolate candy | Short-term | 0000299 | Yes |
| Xanthosine^3^ | Chocolate candy | Habitual | 0000299 | Yes |
| Cyclo(L-prolyl-L-valyl) | Chocolate candy | Short-term  Habitual | 0240493 | Yes |
| Octenoylcarnitine | Vegatbles | Habitual | NA | No |
| 5-HIAA | Fruit | Habitual | 0000763 | No |
| D-Pantothenic acid | Fruit | Habitual | 0000210 | No |
| Hippuric acid^2^ | Fruit | Habitual | 0000714 | Yes |
| Hippuric acid^3^ | Apple | Short-term |  | Yes |
| Ferulic acid 4-O-glucuronide | Orange | Short-term | 0041733 | No |
| Ferulic acid 4-O-sulfate | Orange | Short-term | 0029200 | No |
| Gentisic acid | Potato | Short-term | 0000152 | No |

^1^short-term: dietary intake 1 or 2 days before urine collection; habitual: dietary intake calculated with the United States National Cancer Institute (NCI) method.

^2^measured in positive ionisation mode

^3^measured in negative ionisation mode

**Imputation of missing data**

**Supplementary Table 2 Missing Data Structure**

|  | **W0**  **N = 597** | **W1**  **N = 596** | **W3**  **N = 595** |
| --- | --- | --- | --- |
|  | **Number of missing values N (%)** | | |
| Physical activity | 11 (1.8%) | 16 (2.7%) | 291 (48.9%) |
| HWR z-score | 10 (1.7%) | 0 | 4 (0.7%) |
| CRP z-score | 106 (17.8%) | 85 (14.3%) | 18 (3.0%) |
| Glucose z-score | 59 (9.9%) | 25 (4.2%) | 9 (1.5%) |
| HDL z-score | 41 (6.9%) | 24 (4.0%) | 2 (0.3%) |
| Triglycerides z-score | 42 (7.0%) | 36 (6.0%) | 2 (0.3%) |

HWR, height to weight ratio; CRP, c-reactive protein; HDL, high-density lipoprotein.

Multilevel multiple imputations (n= 20) were performed for HWR z-score, glucose z-score, CRP z-score, HDL z-score, TRG z-score and physical activity. Test runs were conducted to determine the best performing method (2l.pan, 2l.norm or 2l.pmm) for imputation. Further test runs were performed to indicate which (fixed and random) predictors should be incorporated in the final imputation model. Each final imputation model had the education level of the parents, sex and age of the child as fixed effects and the subject ID number as the class variable.

**Supplementary Table 3 methods and predictors for multilevel multiple imputations**

| **Variable** | **Method** | **Fixed effects predictors** | **Random effects predictors** |
| --- | --- | --- | --- |
| Physical activity | 2l.pmm | Country, time point, BMI z-score, HWR z-score | - |
| HWR z-score | 2l.pmm | Country, time point, BMI z-score, physical activity | - |
| CRP z-score | 2l.pmm | Country, time point, BMI z-score, Season of blood collection, glucose z-score, HDL z-score, triglycerides z-score, physical activity | - |
| Glucose z-score | 2l.pan | Season of blood collection | Country, time point, BMI z-score, CRP z-score, HDL z-score, Triglycerides z-score |
| HDL z-score | 2l.pan | Season of blood collection | Country, time point, BMI z-score, Glucose z-score, CRP z-score, Triglycerides z-score |
| Triglycerides z-score | 2l.pmm | Season of blood collection, country, time point, BMI z-score, Glucose z-score, CRP z-score, HDL z-score | Triglycerides z-score |

**Supplementary Table 4 Median concentration of the urine metabolites at each examination wave**

| **Metabolites**^1^ | **W0**  **n=597** | **W1**  **n=596** | **W3**  **n=595** |
| --- | --- | --- | --- |
|  | Median (range) | | |
| Theobromine | 0.23 (-2.50 – 1.93) | 0.25 (-2.50 – 1.72) | 0.20 (-2.50 – 1.80) |
| Xanthosine^2^ | -0.01 (-4.39 – 4.06) | -0.06 (-4.39 – 2.54) | 0.18 (-4.39 – 3.39) |
| Xanthosine^3^ | -0.09 (-4.60 – 3.40) | -0.10 (-4.59 – 3.10) | 0.30 (-5.18 – 3.11) |
| Cyclo(L-prolyl-L-valyl) | 0.26 (-1.94 – 2.42) | 0.17 (-1.94 – 2.21) | 0.33 (-1.94 – 2.12) |
| Octenoylcarnitine | -0.07 (-3.17 – 2.05) | 0.03 (-3.20 – 2.37) | 0.13 (-2.85 – 2.92) |
| 5-HIAA | 0.03 (-5.65 – 5.69) | -0.30 (-5.86 – 3.50) | 0.27 (-5.70 – 3.79) |
| D-Pantothenic acid | 0.26 (-4.08 – 4.41) | 0.08 (-4.28 – 2.57) | -0.32 (-3.23 – 5.42) |
| Hippuric acid^2^ | 0.11 (-5.29 – 2.40) | 0.07 (-5.29 – 2.28) | 0.02 (-5.29 – 2.85) |
| Hippuric acid^3^ | 0.10 (-5.18 – 2.30) | 0.10 (-6.29 – 2.15) | 0.06 (-6.29 – 2.85) |
| Ferulic acid 4-O-glucuronide | 0.12 (-1.21 – 2.69) | 0.06 (-1.21 – 2.53) | 0.13 (-1.21 – 4.10) |
| Ferulic acid 4-O-sulfate | 0.16 (-3.24 – 2.76) | 0.11 (-3.24 – 1.94) | 0.14 (-3.24 – 2.34) |
| Gentisic acid | 0.05 (-1.59 – 3.24) | -0.03 (-1.59 – 3.19) | 0.21 (-1.59 – 2.91) |

^1^Metabolite levels were log-transformed and z-standardised

^2^measured in positive ionisation mode

^3^measured in negative ionisation mode

**Supplementary Table 5 Decomposed explained variance (R^2^) from the linear mixed model for theobromine – 2-year interval**

| **Variable** | **Estimate** | **Lower 95% CI** | **Upper 95% CI** |
| --- | --- | --- | --- |
| Full model | 18.4%^1^ | 16.6% | 24.5% |
| Country | 11.9% | 10% | 18.4% |
| Batch | 0.6% | 0.0% | 7.9% |
| Habitual chocolate intake^2^ | 0.6% | 0.0% | 7.9% |
| Age | 0.8% | 0.0% | 8.1% |
| Sex | 0.6% | 0.0% | 7.9% |
| BMI z-score | 0.1% | 0.0% | 7.4% |
| CRP z-score | 0.0% | 0.0% | 7.3% |
| Glucose z-score | 0.1% | 0.0% | 7.4% |
| HDL z-score | 0.8% | 0.0% | 8.1% |
| TRG z-score | 0.5% | 0.0% | 7.8% |
| Energy intake | 0.0% | 0.0% | 7.3% |
| Parental education level | 0.0% | 0.0% | 7.4% |
| Season of urine collection | 0.4% | 0.0% | 7.7% |
| Time point | 0.3% | 0.0% | 7.6% |

^1^R² from the full model including all variables. This value exceeds the sum of R² values from individual variables, as variables jointly explain shared and additional variance not captured when reported separately.

^2^Habitual: dietary intake calculated with the U.S: National Cancer Institute (NCI) method and n= 599

**Supplementary Table 6 Decomposed explained variance (R^2^) from the linear mixed model for xanthosine measured in positive ionisation mode – 2-year interval**

| **Variable** | **Estimate** | **Lower 95% CI** | **Upper 95% CI** |
| --- | --- | --- | --- |
| Full model | 19.6%^1^ | 17.8% | 28.1% |
| Country | 1.9% | 0.0% | 11.6% |
| Batch | 4.6% | 2.8% | 14.2% |
| Short-term chocolate intake^2^ | 1.5% | 0.0% | 11.3% |
| Age | 0.0% | 0.0% | 9.9% |
| Sex | 0.9% | 0.0% | 10.7% |
| BMI z-score | 3.1% | 1.2% | 12.7% |
| CRP z-score | 1.5% | 0.0% | 11.3% |
| Glucose z-score | 0.0% | 0.0% | 9.9% |
| HDL z-score | 0.0% | 0.0% | 9.9% |
| TRG z-score | 2.1% | 0.2% | 11.8% |
| Energy intake | 0.2% | 0.0% | 10.1% |
| Parental education level | 1.3% | 0.0% | 11.1% |
| Season of urine collection | 0.2% | 0.0% | 10.1% |
| Time point | 0.1% | 0.0% | 10.1% |

^1^R² from the full model including all variables. This value exceeds the sum of R² values from individual variables, as variables jointly explain shared and additional variance not captured when reported separately.

^2^Short-term: dietary intake one or two days prior to urine collection and n= 444

**Supplementary Table 7 Decomposed explained variance (R^2^) from the linear mixed model for xanthosine measured in negative ionisation mode – 2-year interval**

| **Variable** | **Estimate** | **Lower 95% CI** | **Upper 95% CI** |
| --- | --- | --- | --- |
| Full model | 12.4%^1^ | 11.3% | 18.4% |
| Country | 1.0% | 0.0% | 7.6% |
| Batch | 2.2% | 1.1% | 8.7% |
| habitual chocolate intake^2^ | 0.7% | 0.0% | 7.2% |
| Age | 0.0% | 0.0% | 6.6% |
| Sex | 0.6% | 0.0% | 7.1% |
| BMI z-score | 1.6% | 0.5% | 8.1% |
| CRP z-score | 1.0% | 0.0% | 7.5% |
| Glucose z-score | 0.0% | 0.0% | 6.6% |
| HDL z-score | 0.6% | 0.0% | 7.1% |
| TRG z-score | 1.3% | 0.1% | 7.8% |
| Energy intake | 0.5% | 0.0% | 7.0% |
| Parental education level | 0.3% | 0.0% | 6.9% |
| Season of urine collection | 0.6% | 0.0% | 7.1% |
| Time point | 0.0% | 0.0% | 6.6% |

^1^R² from the full model including all variables. This value exceeds the sum of R² values from individual variables, as variables jointly explain shared and additional variance not captured when reported separately.

^2^Habitual: dietary intake calculated with the U.S: National Cancer Institute (NCI) method and n= 599

**Supplementary Table 8 Decomposed explained variance (R^2^) from the linear mixed model for cyclo(L-prolyl-L-valyl) – 2-year interval**

| **Variable** | **Estimate** | **Lower 95% CI** | **Upper 95% CI** |
| --- | --- | --- | --- |
| Full model | 12.8%^1^ | 11.2% | 18.7% |
| Country | 4.5% | 2.9% | 11.0% |
| Batch | 1.3% | 0.0% | 7.9% |
| habitual chocolate intake^2^ | 1.4% | 0.0% | 8.0% |
| Age | 0.1% | 0.0% | 6.8% |
| Sex | 0.0% | 0.0% | 6.7% |
| BMI z-score | 0.0% | 0.0% | 6.7% |
| CRP z-score | 0.1% | 0.0% | 6.7% |
| Glucose z-score | 0.0% | 0.0% | 6.7% |
| HDL z-score | 1.2% | 0.0% | 7.8% |
| TRG z-score | 1.3% | 0.0% | 7.8% |
| Energy intake | 0.1% | 0.0% | 6.7% |
| Parental education level | 0.3% | 0.0% | 7.0% |
| Season of urine collection | 0.3% | 0.0% | 6.9% |
| Time point | 0.1% | 0.0% | 6.8% |

^1^R² from the full model including all variables. This value exceeds the sum of R² values from individual variables, as variables jointly explain shared and additional variance not captured when reported separately.

^2^Habitual: dietary intake calculated with the U.S: National Cancer Institute (NCI) method and n= 599

**Supplementary Table 9 Decomposed explained variance (R^2^) from the linear mixed model for octenoylcarnitine – 2-year interval**

| **Variable** | **Estimate** | **Lower 95% CI** | **Upper 95% CI** |
| --- | --- | --- | --- |
| Full model | 42.4%^1^ | 39.1% | 47.1% |
| Country | 29.2% | 25.8% | 34.8% |
| Batch | 0.8% | 0.0% | 8.6% |
| habitual vegetable intake^2^ | 0.0% | 0.0% | 7.9% |
| Age | 1.0% | 0.0% | 8.8% |
| Sex | 0.0% | 0.0% | 7.9% |
| BMI z-score | 0.0% | 0.0% | 7.9% |
| CRP z-score | 0.1% | 0.0% | 8.0% |
| Glucose z-score | 0.1% | 0.0% | 8.0% |
| HDL z-score | 0.0% | 0.0% | 7.9% |
| TRG z-score | 1.6% | 0.0% | 9.3% |
| Energy intake | 0.0% | 0.0% | 7.9% |
| Parental education level | 0.9% | 0.0% | 8.7% |
| Season of urine collection | 0.1% | 0.0% | 7.9% |
| Time point | 0.1% | 0.0% | 8.0% |

^1^R² from the full model including all variables. This value exceeds the sum of R² values from individual variables, as variables jointly explain shared and additional variance not captured when reported separately.

^2^Habitual: dietary intake calculated with the U.S: National Cancer Institute (NCI) method and n= 599

**Supplementary Table 10 Decomposed explained variance (R^2^) from the linear mixed model for 5-HIAA – 2-year interval**

| **Variable** | **Estimate** | **Lower 95% CI** | **Upper 95% CI** |
| --- | --- | --- | --- |
| Full model | 10.1%^1^ | 9.0% | 15.7% |
| Country | 0.9% | 0.0% | 6.9% |
| Batch | 1.4% | 0.4% | 7.3% |
| habitual fruit intake^2^ | 0.7% | 0.0% | 6.6% |
| Age | 1.0% | 0.1% | 7.0% |
| Sex | 0.0% | 0.0% | 6.0% |
| BMI z-score | 0.3% | 0.0% | 6.3% |
| CRP z-score | 0.1% | 0.0% | 6.1% |
| Glucose z-score | 0.0% | 0.0% | 6.0% |
| HDL z-score | 0.3% | 0.0% | 6.3% |
| TRG z-score | 0.0% | 0.0% | 6.0% |
| Energy intake | 0.0% | 0.0% | 6.0% |
| Parental education level | 0.1% | 0.0% | 6.1% |
| Season of urine collection | 0.4% | 0.0% | 6.4% |
| Time point | 1.3% | 0.3% | 7.2% |

^1^R² from the full model including all variables. This value exceeds the sum of R² values from individual variables, as variables jointly explain shared and additional variance not captured when reported separately.

^2^Habitual: dietary intake calculated with the U.S: National Cancer Institute (NCI) method and n= 599

**Supplementary Table 11 Decomposed explained variance (R^2^) from the linear mixed model for D-Pantothenic acid – 2-year interval**

| **Variable** | **Estimate** | **Lower 95% CI** | **Upper 95% CI** |
| --- | --- | --- | --- |
| Full model | 28.0%^1^ | 25.3% | 33.5% |
| Country | 10.7% | 7.5% | 17.3% |
| Batch | 0.8% | 0.0% | 8.0% |
| habitual fruit intake^2^ | 0.4% | 0.0% | 7.6% |
| Age | 3.1% | 0.0% | 10.3% |
| Sex | 0.1% | 0.0% | 7.3% |
| BMI z-score | 1.1% | 0.0% | 8.3% |
| CRP z-score | 0.6% | 0.0% | 7.8% |
| Glucose z-score | 0.1% | 0.0% | 7.4% |
| HDL z-score | 0.2% | 0.0% | 7.5% |
| TRG z-score | 0.0% | 0.0% | 7.3% |
| Energy intake | 0.0% | 0.0% | 7.3% |
| Parental education level | 0.2% | 0.0% | 7.5% |
| Season of urine collection | 0.0% | 0.0% | 7.3% |
| Time point | 0.0% | 0.0% | 7.1% |

^1^R² from the full model including all variables. This value exceeds the sum of R² values from individual variables, as variables jointly explain shared and additional variance not captured when reported separately.

^2^Habitual: dietary intake calculated with the U.S: National Cancer Institute (NCI) method and n= 599

**Supplementary Table 12 Decomposed explained variance (R^2^) from the linear mixed model for hippuric acid measured in positive ionisation mode – 2-year interval**

| **Variable** | **Estimate** | **Lower 95% CI** | **Upper 95% CI** |
| --- | --- | --- | --- |
| Full model | 24.4%^1^ | 22.1% | 30.5% |
| Country | 11.0% | 8.5% | 17.6% |
| Batch | 1.1% | 0.0% | 8.4% |
| habitual fruit intake^2^ | 0.7% | 0.0% | 8.0% |
| Age | 0.0% | 0.0% | 7.4% |
| Sex | 0.1% | 0.0% | 7.5% |
| BMI z-score | 0.2% | 0.0% | 7.6% |
| CRP z-score | 0.2% | 0.0% | 7.6% |
| Glucose z-score | 0.0% | 0.0% | 7.4% |
| HDL z-score | 0.1% | 0.0% | 7.5% |
| TRG z-score | 0.0% | 0.0% | 7.4% |
| Energy intake | 0.1% | 0.0% | 7.5% |
| Parental education level | 0.0% | 0.0% | 7.4% |
| Season of urine collection | 0.0% | 0.0% | 7.4% |
| Time point | 0.0% | 0.0% | 7.4% |

^1^R² from the full model including all variables. This value exceeds the sum of R² values from individual variables, as variables jointly explain shared and additional variance not captured when reported separately.

^2^Habitual: dietary intake calculated with the U.S: National Cancer Institute (NCI) method and n= 599

**Supplementary Table 13 Decomposed explained variance (R^2^) from the linear mixed model for hippuric acid measured in negative ionisation mode – 2-year interval**

| **Variable** | **Estimate** | **Lower 95% CI** | **Upper 95% CI** |
| --- | --- | --- | --- |
| Full model | 25.0%^1^ | 22.4% | 33.8% |
| Country | 14.7% | 12.2% | 24.1% |
| Batch | 1.9% | 0.0% | 12.5% |
| Short-term apple intake^2^ | 0.1% | 0.0% | 10.8% |
| Age | 0.5% | 0.0% | 11.2% |
| Sex | 0.1% | 0.0% | 10.8% |
| BMI z-score | 0.1% | 0.0% | 10.9% |
| CRP z-score | 0.0% | 0.0% | 10.8% |
| Glucose z-score | 0.1% | 0.0% | 10.9% |
| HDL z-score | 0.0% | 0.0% | 10.8% |
| TRG z-score | 0.0% | 0.0% | 10.8% |
| Energy intake | 0.0% | 0.0% | 10.8% |
| Parental education level | 0.1% | 0.0% | 10.8% |
| Season of urine collection | 0.4% | 0.0% | 11.1% |
| Time point | 0.1% | 0.0% | 10.9% |

^1^R² from the full model including all variables. This value exceeds the sum of R² values from individual variables, as variables jointly explain shared and additional variance not captured when reported separately.

^2^Short-term: dietary intake one or two days prior to urine collection and n= 444

**Supplementary Table 14 Decomposed explained variance (R^2^) from the linear mixed model for ferulic acid 4-O-glucuronide – 2-year interval**

| **Variable** | **Estimate** | **Lower 95% CI** | **Upper 95% CI** |
| --- | --- | --- | --- |
| Full model | 15.7%^1^ | 14.2% | 25.1% |
| Country | 5.5% | 3.6% | 15.2% |
| Batch | 1.6% | 0.0% | 11.7% |
| Short-term orange intake^2^ | 0.6% | 0.0% | 10.8% |
| Age | 1.1% | 0.0% | 11.3% |
| Sex | 1.0% | 0.0% | 11.2% |
| BMI z-score | 0.6% | 0.0% | 10.8% |
| CRP z-score | 0.3% | 0.0% | 10.6% |
| Glucose z-score | 0.8% | 0.0% | 11.0% |
| HDL z-score | 0.2% | 0.0% | 10.5% |
| TRG z-score | 0.0% | 0.0% | 10.3% |
| Energy intake | 0.1% | 0.0% | 10.3% |
| Parental education level | 0.1% | 0.0% | 10.4% |
| Season of urine collection | 0.3% | 0.0% | 10.6% |
| Time point | 1.5% | 0.0% | 11.7% |

^1^R² from the full model including all variables. This value exceeds the sum of R² values from individual variables, as variables jointly explain shared and additional variance not captured when reported separately.

^2^Short-term: dietary intake one or two days prior to urine collection and n= 444

**Supplementary Table 15 Decomposed explained variance (R^2^) from the linear mixed model for ferulic acid 4-O-sulfate – 2-year interval**

| **Variable** | **Estimate** | **Lower 95% CI** | **Upper 95% CI** |
| --- | --- | --- | --- |
| Full model | 10.8%^1^ | 10.6% | 20.2% |
| Country | 2.6% | 2.6% | 12.5% |
| Batch | 1.4% | 1.4% | 11.4% |
| Short-term orange intake^2^ | 1.3% | 1.3% | 11.3% |
| Age | 0.1% | 0.1% | 10.1% |
| Sex | 0.1% | 0.2% | 10.2% |
| BMI z-score | 0.7% | 0.7% | 10.7% |
| CRP z-score | 0.8% | 0.8% | 10.8% |
| Glucose z-score | 0.4% | 0.4% | 10.4% |
| HDL z-score | 0.2% | 0.2% | 10.3% |
| TRG z-score | 0.2% | 0.2% | 10.2% |
| Energy intake | 0.2% | 0.2% | 10.2% |
| Parental education level | 0.0% | 0.0% | 10.0% |
| Season of urine collection | 0.3% | 0.3% | 10.3% |
| Time point | 0.2% | 0.2% | 10.2% |

^1^R² from the full model including all variables. This value exceeds the sum of R² values from individual variables, as variables jointly explain shared and additional variance not captured when reported separately.

^2^Short-term: dietary intake one or two days prior to urine collection and n= 444

**Supplementary Table 16 Decomposed explained variance (R^2^) from the linear mixed model for gentisic acid – 2-year interval**

| **Variable** | **Estimate** | **Lower 95% CI** | **Upper 95% CI** |
| --- | --- | --- | --- |
| Full model | 9.8%^1^ | 9.5% | 18.5% |
| Country | 3.5% | 3.3% | 12.6% |
| Batch | 1.2% | 0.9% | 10.5% |
| Short-term potatoe intake^2^ | 1.5% | 1.3% | 10.8% |
| Age | 0.1% | 0.0% | 9.4% |
| Sex | 0.2% | 0.0% | 9.6% |
| BMI z-score | 0.5% | 0.2% | 9.9% |
| CRP z-score | 0.3% | 0.0% | 9.7% |
| Glucose z-score | 0.1% | 0.0% | 9.5% |
| HDL z-score | 0.0% | 0.0% | 9.4% |
| TRG z-score | 0.3% | 0.0% | 9.7% |
| Energy intake | 0.0% | 0.0% | 9.4% |
| Parental education level | 0.2% | 0.0% | 9.6% |
| Season of urine collection | 0.2% | 0.0% | 9.6% |
| Time point | 0.1% | 0.0% | 9.4% |

^1^R² from the full model including all variables. This value exceeds the sum of R² values from individual variables, as variables jointly explain shared and additional variance not captured when reported separately.

^2^Short-term: dietary intake one or two days prior to urine collection and n= 444

**Supplementary Table 17 Decomposed explained variance (R^2^) from the linear mixed model for theobromine – 4-year interval**

| **Variable** | **Estimate** | **Lower 95% CI** | **Upper 95% CI** |
| --- | --- | --- | --- |
| Full model | 15.2%^1^ | 13.4% | 20.7% |
| Country | 10.2% | 8.5% | 16.0% |
| Batch | 0.5% | 0.0% | 6.8% |
| habitual chocolate intake^2^ | 0.6% | 0.0% | 6.9% |
| Age | 0.6% | 0.0% | 6.8% |
| Sex | 0.6% | 0.0% | 6.9% |
| BMI z-score | 0.0% | 0.0% | 6.4% |
| CRP z-score | 0.0% | 0.0% | 6.3% |
| Glucose z-score | 0.0% | 0.0% | 6.3% |
| HDL z-score | 0.5% | 0.0% | 6.8% |
| TRG z-score | 0.5% | 0.0% | 6.8% |
| Energy intake | 0.0% | 0.0% | 6.4% |
| Parental education level | 0.1% | 0.0% | 6.4% |
| Season of urine collection | 0.1% | 0.0% | 6.5% |
| Time point | 0.4% | 0.0% | 6.7% |

^1^R² from the full model including all variables. This value exceeds the sum of R² values from individual variables, as variables jointly explain shared and additional variance not captured when reported separately.

^2^Habitual: dietary intake calculated with the U.S: National Cancer Institute (NCI) method and n= 599

**Supplementary Table 18 Decomposed explained variance (R^2^) from the linear mixed model for xanthosine measured in positive ionisation mode – 4-year interval**

| **Variable** | **Estimate** | **Lower 95% CI** | **Upper 95% CI** |
| --- | --- | --- | --- |
| Full model | 19.0%^1^ | 17.2% | 26.0% |
| Country | 2.2% | 0.1% | 10.9% |
| Batch | 5.1% | 3.1% | 13.5% |
| Short-term chocolate intake^2^ | 1.0% | 0.0% | 9.7% |
| Age | 0.8% | 0.0% | 9.6% |
| Sex | 0.7% | 0.0% | 9.5% |
| BMI z-score | 2.6% | 0.4% | 11.2% |
| CRP z-score | 0.2% | 0.0% | 9.0% |
| Glucose z-score | 0.0% | 0.0% | 8.9% |
| HDL z-score | 0.0% | 0.0% | 8.9% |
| TRG z-score | 1.9% | 0.0% | 10.6% |
| Energy intake | 0.4% | 0.0% | 9.3% |
| Parental education level | 0.7% | 0.0% | 9.5% |
| Season of urine collection | 0.0% | 0.0% | 8.9% |
| Time point | 0.6% | 0.0% | 9.4% |

^1^R² from the full model including all variables. This value exceeds the sum of R² values from individual variables, as variables jointly explain shared and additional variance not captured when reported separately.

^2^Short-term: dietary intake one or two days prior to urine collection and n= 444

**Supplementary Table 19 Decomposed explained variance (R^2^) from the linear mixed model for xanthosine measured in negative ionisation mode – 4-year interval**

| **Variable** | **Estimate** | **Lower 95% CI** | **Upper 95% CI** |
| --- | --- | --- | --- |
| Full model | 14.1%^1^ | 12.4% | 19.3% |
| Country | 1.2% | 0.0% | 6.9% |
| Batch | 1.9% | 0.1% | 7.6% |
| habitual chocolate intake^2^ | 0.5% | 0.0% | 6.1% |
| Age | 0.6% | 0.0% | 6.3% |
| Sex | 0.7% | 0.0% | 6.4% |
| BMI z-score | 1.5% | 0.0% | 7.2% |
| CRP z-score | 0.1% | 0.0% | 5.8% |
| Glucose z-score | 0.0% | 0.0% | 5.7% |
| HDL z-score | 0.4% | 0.0% | 6.1% |
| TRG z-score | 0.7% | 0.0% | 6.4% |
| Energy intake | 0.5% | 0.0% | 6.2% |
| Parental education level | 0.3% | 0.0% | 6.0% |
| Season of urine collection | 0.3% | 0.0% | 6.0% |
| Time point | 0.3% | 0.0% | 6.0% |

^1^R² from the full model including all variables. This value exceeds the sum of R² values from individual variables, as variables jointly explain shared and additional variance not captured when reported separately.

^2^Habitual: dietary intake calculated with the U.S: National Cancer Institute (NCI) method and n= 599

**Supplementary Table 20 Decomposed explained variance (R^2^) from the linear mixed model for cyclo(L-prolyl-L-valyl) – 4-year interval**

| **Variable** | **Estimate** | **Lower 95% CI** | **Upper 95% CI** |
| --- | --- | --- | --- |
| Full model | 10.8%^1^ | 9.6% | 15.7% |
| Country | 4.5% | 3.3% | 9.6% |
| Batch | 1.5% | 0.3% | 6.7% |
| habitual chocolate intake^2^ | 1.0% | 0.0% | 6.3% |
| Age | 0.2% | 0.0% | 5.5% |
| Sex | 0.0% | 0.0% | 5.4% |
| BMI z-score | 0.0% | 0.0% | 5.4% |
| CRP z-score | 0.0% | 0.0% | 5.3% |
| Glucose z-score | 0.0% | 0.0% | 5.3% |
| HDL z-score | 0.7% | 0.0% | 6.0% |
| TRG z-score | 0.7% | 0.0% | 6.1% |
| Energy intake | 0.1% | 0.0% | 5.4% |
| Parental education level | 0.0% | 0.0% | 5.4% |
| Season of urine collection | 0.1% | 0.0% | 5.4% |
| Time point | 0.2% | 0.0% | 5.5% |

^1^R² from the full model including all variables. This value exceeds the sum of R² values from individual variables, as variables jointly explain shared and additional variance not captured when reported separately.

^2^Habitual: dietary intake calculated with the U.S: National Cancer Institute (NCI) method and n= 599

**Supplementary Table 21 Decomposed explained variance (R^2^) from the linear mixed model for octenoylcarnitine – 4-year interval**

| **Variable** | **Estimate** | **Lower 95% CI** | **Upper 95% CI** |
| --- | --- | --- | --- |
| Full model | 43.8%^1^ | 41.0% | 47.6% |
| Country | 30.5% | 27.1% | 34.8% |
| Batch | 0.9% | 0.0% | 7.1% |
| habitual vegetable intake^2^ | 0.0% | 0.0% | 6.3% |
| Age | 0.3% | 0.0% | 6.6% |
| Sex | 0.0% | 0.0% | 6.3% |
| BMI z-score | 0.1% | 0.0% | 6.4% |
| CRP z-score | 0.1% | 0.0% | 6.4% |
| Glucose z-score | 0.0% | 0.0% | 6.3% |
| HDL z-score | 0.0% | 0.0% | 6.3% |
| TRG z-score | 1.5% | 0.0% | 7.7% |
| Energy intake | 0.0% | 0.0% | 6.3% |
| Parental education level | 0.3% | 0.0% | 6.6% |
| Season of urine collection | 0.2% | 0.0% | 6.5% |
| Time point | 0.0% | 0.0% | 6.3% |

^1^R² from the full model including all variables. This value exceeds the sum of R² values from individual variables, as variables jointly explain shared and additional variance not captured when reported separately.

^2^Habitual: dietary intake calculated with the U.S: National Cancer Institute (NCI) method and n= 599

**Supplementary Table 22 Decomposed explained variance (R^2^) from the linear mixed model for 5-HIAA – 4-year interval**

| **Variable** | **Estimate** | **Lower 95% CI** | **Upper 95% CI** |
| --- | --- | --- | --- |
| Full model | 13.9%^1^ | 12.8% | 18.5% |
| Country | 0.5% | 0.0% | 5.4% |
| Batch | 1.8% | 0.6% | 6.7% |
| habitual fruit intake^2^ | 0.8% | 0.0% | 5.7% |
| Age | 0.8% | 0.0% | 5.7% |
| Sex | 0.1% | 0.0% | 5.0% |
| BMI z-score | 0.6% | 0.0% | 5.5% |
| CRP z-score | 0.1% | 0.0% | 5.0% |
| Glucose z-score | 0.0% | 0.0% | 4.9% |
| HDL z-score | 0.1% | 0.0% | 5.0% |
| TRG z-score | 0.1% | 0.0% | 5.0% |
| Energy intake | 0.0% | 0.0% | 4.9% |
| Parental education level | 0.1% | 0.0% | 5.0% |
| Season of urine collection | 0.3% | 0.0% | 5.2% |
| Time point | 7.8% | 6.6% | 12.5% |

^1^R² from the full model including all variables. This value exceeds the sum of R² values from individual variables, as variables jointly explain shared and additional variance not captured when reported separately.

^2^Habitual: dietary intake calculated with the U.S: National Cancer Institute (NCI) method and n= 599

**Supplementary Table 23 Decomposed explained variance (R^2^) from the linear mixed model for D-Pantothenic – 4-year interval acid**

| **Variable** | **Estimate** | **Lower 95% CI** | **Upper 95% CI** |
| --- | --- | --- | --- |
| Full model | 28.5%^1^ | 26.2% | 33.3% |
| Country | 8.9% | 6.1% | 14.5% |
| Batch | 0.7% | 0.0% | 6.7% |
| habitual fruit intake^2^ | 0.4% | 0.0% | 6.5% |
| Age | 3.3% | 0.3% | 9.2% |
| Sex | 0.2% | 0.0% | 6.3% |
| BMI z-score | 1.0% | 0.0% | 7.0% |
| CRP z-score | 0.3% | 0.0% | 6.4% |
| Glucose z-score | 0.1% | 0.0% | 6.2% |
| HDL z-score | 0.0% | 0.0% | 6.1% |
| TRG z-score | 0.0% | 0.0% | 6.1% |
| Energy intake | 0.0% | 0.0% | 6.1% |
| Parental education level | 0.2% | 0.0% | 6.3% |
| Season of urine collection | 0.0% | 0.0% | 6.1% |
| Time point | 0.5% | 0.0% | 6.5% |

^1^R² from the full model including all variables. This value exceeds the sum of R² values from individual variables, as variables jointly explain shared and additional variance not captured when reported separately.

^2^Habitual: dietary intake calculated with the U.S: National Cancer Institute (NCI) method and n= 599

**Supplementary Table 24 Decomposed explained variance (R^2^) from the linear mixed model for hippuric acid measured in positive ionisation mode – 4-year interval acid**

| **Variable** | **Estimate** | **Lower 95% CI** | **Upper 95% CI** |
| --- | --- | --- | --- |
| Full model | 22.7%^1^ | 20.6% | 27.5% |
| Country | 10.9% | 8.8% | 16.2% |
| Batch | 0.9% | 0.0% | 6.6% |
| habitual fruit intake^2^ | 0.6% | 0.0% | 6.3% |
| Age | 0.0% | 0.0% | 5.7% |
| Sex | 0.1% | 0.0% | 5.8% |
| BMI z-score | 0.2% | 0.0% | 5.9% |
| CRP z-score | 0.2% | 0.0% | 5.9% |
| Glucose z-score | 0.0% | 0.0% | 5.7% |
| HDL z-score | 0.1% | 0.0% | 5.8% |
| TRG z-score | 0.0% | 0.0% | 5.7% |
| Energy intake | 0.0% | 0.0% | 5.8% |
| Parental education level | 0.0% | 0.0% | 5.8% |
| Season of urine collection | 0.1% | 0.0% | 5.8% |
| Time point | 0.1% | 0.0% | 5.8% |

^1^R² from the full model including all variables. This value exceeds the sum of R² values from individual variables, as variables jointly explain shared and additional variance not captured when reported separately.

^2^Habitual: dietary intake calculated with the U.S: National Cancer Institute (NCI) method and n= 599

**Supplementary Table 25 Decomposed explained variance (R^2^) from the linear mixed model for hippuric acid measured in negative ionisation mode – 4-year interval acid**

| **Variable** | **Estimate** | **Lower 95% CI** | **Upper 95% CI** |
| --- | --- | --- | --- |
| Full model | 20.1%^1^ | 17.6% | 27.4% |
| Country | 11.7% | 9.2% | 19.3% |
| Batch | 1.6% | 0.0% | 10.0% |
| Short-term apple intake^2^ | 0.0% | 0.0% | 8.5% |
| Age | 0.2% | 0.0% | 8.6% |
| Sex | 0.2% | 0.0% | 8.6% |
| BMI z-score | 0.2% | 0.0% | 8.6% |
| CRP z-score | 0.0% | 0.0% | 8.5% |
| Glucose z-score | 0.1% | 0.0% | 8.5% |
| HDL z-score | 0.0% | 0.0% | 8.4% |
| TRG z-score | 0.0% | 0.0% | 8.4% |
| Energy intake | 0.0% | 0.0% | 8.5% |
| Parental education level | 0.0% | 0.0% | 8.5% |
| Season of urine collection | 0.6% | 0.0% | 9.0% |
| Time point | 0.0% | 0.0% | 8.5% |

^1^R² from the full model including all variables. This value exceeds the sum of R² values from individual variables, as variables jointly explain shared and additional variance not captured when reported separately.

^2^Short-term: dietary intake one or two days prior to urine collection and n= 444

**Supplementary Table 26 Decomposed explained variance (R^2^) from the linear mixed model for ferulic acid 4-O-glucuronide – 4-year interval acid**

| **Variable** | **Estimate** | **Lower 95% CI** | **Upper 95% CI** |
| --- | --- | --- | --- |
| Full model | 11.6%^1^ | 10.3% | 19.4% |
| Country | 4.4% | 3.5% | 12.6% |
| Batch | 1.2% | 0.2% | 9.5% |
| Short-term orange intake^2^ | 0.1% | 0.0% | 8.5% |
| Age | 0.6% | 0.0% | 9.0% |
| Sex | 1.5% | 0.5% | 9.8% |
| BMI z-score | 0.2% | 0.0% | 8.7% |
| CRP z-score | 0.2% | 0.0% | 8.6% |
| Glucose z-score | 0.6% | 0.0% | 9.0% |
| HDL z-score | 0.1% | 0.0% | 8.6% |
| TRG z-score | 0.1% | 0.0% | 8.5% |
| Energy intake | 0.0% | 0.0% | 8.5% |
| Parental education level | 0.1% | 0.0% | 8.5% |
| Season of urine collection | 0.3% | 0.0% | 8.7% |
| Time point | 0.8% | 0.0% | 9.1% |

^1^R² from the full model including all variables. This value exceeds the sum of R² values from individual variables, as variables jointly explain shared and additional variance not captured when reported separately.

^2^Short-term: dietary intake one or two days prior to urine collection and n= 444

**Supplementary Table 27 Decomposed explained variance (R^2^) from the linear mixed model for ferulic acid 4-O-sulfate – 4-year interval acid**

| **Variable** | **Estimate** | **Lower 95% CI** | **Upper 95% CI** |
| --- | --- | --- | --- |
| Full model | 8.3%^1^ | 7.9% | 16.1% |
| Country | 2.8% | 2.5% | 10.9% |
| Batch | 0.9% | 0.7% | 9.2% |
| Short-term orange intake^2^ | 0.7% | 0.5% | 9.0% |
| Age | 0.2% | 0.0% | 8.6% |
| Sex | 0.5% | 0.2% | 8.8% |
| BMI z-score | 0.4% | 0.2% | 8.7% |
| CRP z-score | 0.2% | 0.0% | 8.5% |
| Glucose z-score | 0.3% | 0.1% | 8.6% |
| HDL z-score | 0.0% | 0.0% | 8.4% |
| TRG z-score | 0.0% | 0.0% | 8.4% |
| Energy intake | 0.1% | 0.0% | 8.4% |
| Parental education level | 0.0% | 0.0% | 8.3% |
| Season of urine collection | 0.1% | 0.0% | 8.5% |
| Time point | 0.2% | 0.0% | 8.6% |

^1^R² from the full model including all variables. This value exceeds the sum of R² values from individual variables, as variables jointly explain shared and additional variance not captured when reported separately.

^2^Short-term: dietary intake one or two days prior to urine collection and n= 444

**Supplementary Table 28 Decomposed explained variance (R^2^) from the linear mixed model for gentisic acid – 4-year interval acid**

| **Variable** | **Estimate** | **Lower 95% CI** | **Upper 95% CI** |
| --- | --- | --- | --- |
| Full model | 11.0%^1^ | 10.4% | 18.2% |
| Country | 4.4% | 3.9% | 12.0% |
| Batch | 1.2% | 0.7% | 9.1% |
| Short-term potatoe intake^2^ | 1.1% | 0.6% | 9.0% |
| Age | 0.0% | 0.0% | 8.0% |
| Sex | 0.2% | 0.0% | 8.2% |
| BMI z-score | 0.3% | 0.0% | 8.3% |
| CRP z-score | 0.0% | 0.0% | 8.0% |
| Glucose z-score | 0.1% | 0.0% | 8.0% |
| HDL z-score | 0.0% | 0.0% | 8.0% |
| TRG z-score | 0.1% | 0.0% | 8.1% |
| Energy intake | 0.1% | 0.0% | 8.0% |
| Parental education level | 0.3% | 0.0% | 8.2% |
| Season of urine collection | 0.2% | 0.0% | 8.1% |
| Time point | 0.4% | 0.0% | 8.3% |

^1^R² from the full model including all variables. This value exceeds the sum of R² values from individual variables, as variables jointly explain shared and additional variance not captured when reported separately.

^2^Short-term: dietary intake one or two days prior to urine collection and n= 444

**References**

1. Intemann, T., et al., *Urinary sucrose and fructose to validate self-reported sugar intake in children and adolescents: results from the I. Family study.* European journal of nutrition, 2019. **58**(3): p. 1247-1258.

2. Hebestreit, A., et al., *Cross-sectional and longitudinal associations between energy intake and BMI z-score in European children.* International Journal of Behavioral Nutrition and Physical Activity, 2016. **13**(1): p. 1-11.

3. Hebestreit, A., et al., *Dietary patterns of European children and their parents in association with family food environment: Results from the I. family study.* Nutrients, 2017. **9**(2): p. 126.

4. Hebestreit, A., et al., *Web-based 24-h dietary recall: the SACANA program*, in *Instruments for health surveys in children and adolescents*. 2019, Springer. p. 77-102.

5. Iglesia, I., et al., *Dairy Consumption at Snack Meal Occasions and the Overall Quality of Diet during Childhood. Prospective and Cross-Sectional Analyses from the IDEFICS/I.Family Cohort.* Nutrients, 2020. **12**(3): p. 642.

6. Huybrechts, I., et al., *Evaluation of the Children's Eating Habits Questionnaire used in the IDEFICS study by relating urinary calcium and potassium to milk consumption frequencies among European children.* International journal of Obesity, 2011. **35**(1): p. S69-S78.

7. Goerdten, J., et al., *Identification and replication of urine metabolites associated with short-term and habitual intake of sweet and fatty snacks in European children and adolescents.* The Journal of Nutrition, 2024. **154**(11): p. 3274-3285.

8. Buyken, A., et al., *Die DONALD Kohorte.* Bundesgesundheitsblatt-Gesundheitsforschung-Gesundheitsschutz, 2012. **55**(6-7): p. 875-884.
